# Local Volume Concentration, Packing Domains and Scaling Properties of Chromatin

**DOI:** 10.21203/rs.3.rs-3399177/v1

**Published:** 2023-10-17

**Authors:** Marcelo Carignano, Martin Kröger, Luay Almassalha, Vasundhara Agrawal, Wing Shun Li, Emily M. Pujadas, Rikkert J. Nap, Vadim Backman, Igal Szleifer

**Affiliations:** 1 Department of Biomedical Engineering, Northwestern University, Evanston, IL 60208, USA.; 2 Magnetism and Interface Physics & Computational Polymer Physics, Department of Materials, ETH Zurich, CH-8093 Zurich, Switzerland.; 3 Applied Physics Program, Northwestern University, Evanston, IL 60208, USA.; 4 Department of Gastroenterology and Hepatology, Northwestern Memorial Hospital, Chicago IL 60611, USA.; 5 Department of Chemistry, Northwestern University, Evanston, IL 60208, USA.; 6 These authors contributed equally: Marcelo Carignano. Martin Kröger and Luay Almassalha

## Abstract

We propose the Self Returning Excluded Volume (SR-EV) model for the structure of chromatin based on stochastic rules and physical interactions that is able to capture the observed behavior across imaging and sequencing based measures of chromatin organization. The SR-EV model takes the return rules of the Self Returning Random Walk, incorporates excluded volume interactions, chain connectivity and expands the length scales range from 10 nm to over 1 micron. The model is computationally fast and we created thousands of configurations that we grouped in twelve different ensembles according to the two main parameters of the model. The analysis of the configurations was done in a way completely analogous to the experimental treatments used to determine chromatin volume concentration, contact probability, packing domain identification and size characterization, and packing scaling behavior. We find a robust agreement between the theoretical and experimental results. The overall organization of the model chromatin is corrugated, with dense packing domains alternating with a very dilute regions in a manner that resembles the mixing of two disordered bi-continuous phases. The return rules combined with excluded volume interactions lead to the formation of packing domains. We observed a transition from a short scale regime to a long scale regime occurring at genomic separations of ~ 4 × 10^4^ base pairs or ~ 100 nm in distance. The contact probability reflects this transition with a change in the scaling exponent from larger than −1 to approximately −1. The analysis of the pair correlation function reveals that chromatin organizes following a power law scaling with exponent D∈{2,3} in the transition region between the short and long distance regimes.

## Introduction

Chromatin is a complex macromolecular fiber that results from the assembly of DNA with histone and non-histone proteins to form the functional organization of the genome within the eukaryotic cell nucleus. That over 2-linear meters (~ 3 × 10^9^ base pairs) is confined within human nuclei ranging between 5 to 10 nm in diameter while maintaining functionally relevant information creates a core dilemma that places a tension between efficiency of packing with information retention (1). Adding to this complexity are the rich heterogeneity of non-chromatin nuclear bodies, histone concentrations within normal cells, and chromosome copy number (and total DNA content) in malignant cells (2–5). Despite the profound degree of variability from cell-to-cell even within microscopically normal tissues (6), the ensemble function of organs is maintained by facilitating the preferential activation of specific gene network patterns. In these contexts, describing chromatin as a stochasticaly evolving process with constraints appears to be a rational approach to represent the regulatory processes that couple structure with function (7). Unfortunately, at present no such functionally-appropriate statistical framework describing chromatin organization exists.

There are many important efforts to model chromatin from an atomistic or a nearly atomistic approach addressing different processes involving DNA, histones and other proteins (8–29). From the other end of the chromatin length scale the aim is to use experimental results, especially from high-throughput chromatin conformation capture (Hi-C) (30), to guide polymer models simulations with especial characteristics that can replicate for example, contact patterns and loop extrusion process. (31–39) Many lines of evidence support the idea of chromatin configurations as a statistical assembly that produce functional organization. First, the overwhelming majority of the genome does not code for proteins but has functional consequences at the level of regulating gene transcription. Second, Hi-C (30) and similar techniques identify the presence of compartments, domains, and loops; however, these structures only become evident as distinct contact loci with millions of sequence measurements (40, 41). Third, single cell sequencing and *in situ* sequencing of normal tissue and malignancies has demonstrated profound heterogeneity in transcriptional patterns that were previously not appreciated under routine histological examination (4). Finally, ongoing methods investigating chromatins structure have shown that it is dynamically evolving even at the order of seconds to minutes (42).

Therefore, in this work, we address this fundamental gap in knowledge by proposing a minimal model based purely on molecular, physical, and statistical principles which *i*) preserves the efficiency of chromatin packing, *ii*) produces the observed structural heterogeneity and population diversity, *iii*) retains the capacity for functionally relevant storage of genomic information. We begin by assuming that there is an overall statistical rule governing the spatial organization of chromatin. Inspired by the known features of genomic structure, we demonstrate that these processes are produced by the interplay between low-frequency large extrusion-returns and the excluded volume of monomeric units (e.g. nucleosomes). In this work, we focus not on demonstrating the ability to define structure of particular loci, but to provide evidence that a statistically-grounded method achieves the experimentally observed structure within cell nuclei. We compare our model directly with the observed, ground truth 3D structure of the mammalian genome observed via Chromatin Scanning Transmission Electron Microscopy (ChromSTEM). As expected, our model produces the 3D features of genomic organization observed under ChromSTEM without requiring arbitrary parameter fitting. Both in our model and experimentally, irregular assemblies of fibers with a radius of 60 nm are produced while also achieving the widely observed average nuclear density of 20–30%.

Further, in agreement with ChromSTEM and many other experimental methods, we find that genomic folding has a characteristic radial dependency that can be interpreted in terms of a power-law with an exponent of D. To understand if this power-law structure observed within our model extends into live cells, we performed experiments utilizing live-cell Partial Wave Spectroscopic (PWS) microscopy (43, 44) that can measure the power-law scaling of genome structure without the use of exogenous labels. We found that our minimal model both represented the power-law structure of chromatin and the level of diversity found within the cellular population. The structures predicted by our model display a porosity that result from the alternation of high and low density regions. The envelope of the high density regions could be regarded as the separating interphase of a bi-continuous system that is a topological scenario that favors extensive mobility of proteins, mRNA and other free crowders while providing a large accessible surface area of chromatin. The contact probability, calculated as an ensemble average, shows a good agreement with Hi-C results displaying a transition between intra-domain and inter-domain regimes. The intra-domain contact probability scales with an exponent larger than −1, while the inter-domain one scales with an exponent similar to −1. As such, this work introduces the basis for a statistical representation of the genome structure.

### A Minimal Model for Chromatin Conformations

The Self Returning-Excluded Volume (SR-EV) model for chromatin is derived from the Self Returning Random Walk (SRRW) model that was recently introduced by this group (45). Here, we review the SRRW model and then we introduce the modifications that lead to the SR-EV model.

The SRRW model is essentially a random walk with specific rules introduced to capture statistical features of chromatin organization as revealed by experiments. At each step in the SRRW generation there are two possibilities: i) Perform a forward jump or ii) Return over the previous step to the previous position. The probability $P_{R}$ for a return step is given by

(1) $$P_{R}\left(U_{0}\right)=\frac{U_{0}^{-\alpha }}{\alpha }.$$

Here, $U_{0}$ is the length of the last step along the backbone over which the walk may return. The folding parameter α>1 controls the number of returns. If the SRRW does not continue with a return step, it must continue with a forward jump. The new forward jump is chosen with an random direction and with a length $U_{1}$ given by the following probability distribution function (pdf)

(2) $$P_{J}\left(U_{1}>1\right)=\frac{\alpha \;+\;1}{U_{1}^{\alpha +2}}$$

We will generally refer to Eqs. (1) and (2) as the *return rules* of the SR-EV model. There is a minimum size for the forward jumps that also defines the unit of length in the model. The succession of forward jumps and return steps leads to a structure than can be regarded as a linear backbone with tree-like branches along its length, with the branching points representing overlaps created by the return steps. In addition to the return probability and pdf defined above, the SRRW generation algorithm (contained in the Supplementary Information) includes a local cutoff to avoid unrealistically long steps and a spherical global cutoff to contain the configuration. The global cutoff is applied during the generation of the conformation and is measured from the center of mass of the already-generated steps.

**Figure 1:**
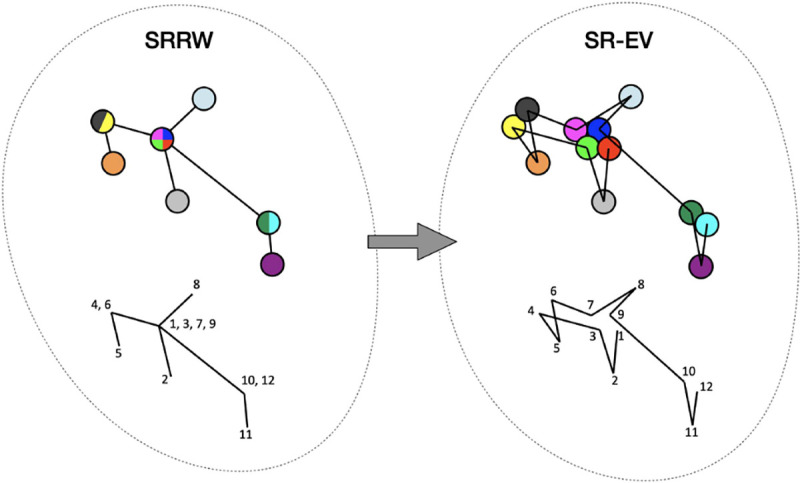
Schematic representation of the conversion process from SRRW to SR-EV. The SRRW configurational motif hides the overlap of several beads in a molecule that has the structure of a branching polymer. By the introduction of excluded volume in SR-EV, the overlapping beads separate to form a cluster and a linear molecule.

**Figure 2:**
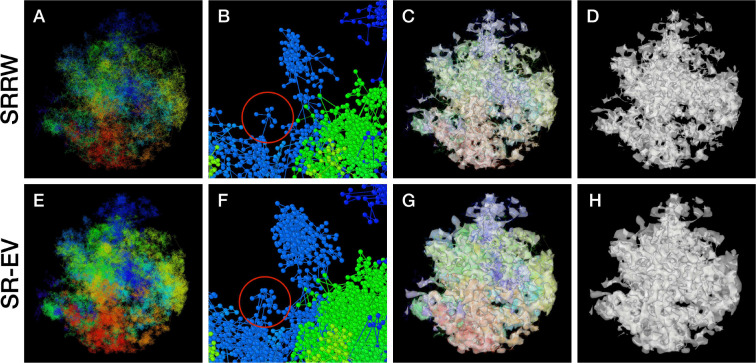
Example SRRW and SR-EV configurations. The top row are for the SRRW case, and bottom row corresponds to the associated SR-EV configuration. (A) and (E) represent the bonds of the full configurations and show that while SR-EV looks denser than the SRRW case the overall structure is preserved upon removal of the original overlaps. (B) and (F) correspond to the same small portion of the conformation and shows SR-EV having many more beads than SRRW due to the excluded volume between beads. The red circles explicitly highlight a structural motif that in SRRW is a central bead with 7 bonds branching out (a sequence of seven consecutive jump and returns steps) that transform to 15 linearly connecting beads forming a cluster. (C) and (G) display the chromatin conformations wrapped by a tight mesh suggesting the separation between a chromatin rich and a chromatin depleted regions, the latter being the space that free crowders could easily occupy. (D) and (H) show the bare interface between the two regions that resembles the interface dividing two bi-continuous phases and also clearly expose the difference between SRRW and SR-EV.

By construction, since the SRRW includes returns over the previous steps, it contains a large number of overlaps. For α=1.10,1.15, and 1.20 the number of returns is 48.7%, 47.5%, and 46.2% of the total number of steps, respectively. Therefore, as a representation of a physical system, such as chromatin, the SRRW has two important drawbacks: i) the conformations violate the principle of excluded volume and ii) it is not a linear polymer. In order to recover these two physical properties we extended the SRRW to develop the SR-EV model. In this new method, the overlapping points are transformed into connected clusters of beads that explicitly represent a linear chain, as shown on the scheme displayed in Figure 1. The method that we employ to remove overlaps is a low-temperature-controlled molecular dynamics simulation using a soft repulsive interaction potential between initially overlapping beads, that is terminated as soon as all overlaps have been resolved, as described in the Supplementary Information. An example of an SRRW configuration and its corresponding SR-EV are displayed on Figure 2-A and 2-E, respectively. Figures 2-B and 2-F represent a small region on the periphery of the configuration and exemplifies how structures formed by a sequence of forward and returns steps expands to a larger cluster after including excluded volume interactions. The porosity of the structure is also affected by the excluded volume introduced in SR-EV.

For this work we adopted a *unit length* of 10 nm, similar to the diameter of a nucleosome (46). Therefore, each bead of the model chromatin represents a nucleosome. The spherical global cutoff was set to $R_{c}=650\;nm$. From the resulting conformations we can cut slabs spanning well over 1 μm in cross section. Excluded volume was introduced by imposing a non overlap radius of $r_{\circ }=4.9\;nm$ between all the beads of the SR-EV model. With these quantities, we defined the overall average volume fraction as $\phi =N{\left(r_{\circ }/R_{c}\right)}^{3}$, with N the number of beads in the chromatin model chain. We considered four different volume fractions ϕ=0.08,0.12,0.16 and 0.20, which correspond to N=186741,280112,373483 and 466 854, respectively. Each one of these four average volume fractions were studied with three different folding parameters α=1.10,1.15 and 1.20. SR-EV configurations, as we present them in this work, are associated to the structure of a single chromosome. Therefore, all the analysis that follows is done on the structure of a single chromosome system. For each combination of ϕ and α we created an ensemble of 1 000 different chromatin configurations. In order to introduce the genomic distance along the SR-EV configuration we assign 147 base pairs to each nucleosome, representing the length of DNA wrapping the histone octamers. Considering that the effective bead diameter is 9.8 nm, the average distance between adjacent base pairs in the DNA double helix, and the model bonds $U_{i}$ that are larger than 10 nm, we assign the number of base pairs in the linker DNA as the nearest integer of $\left(U_{i}-9.8\;nm\right)/(0.34\;nm)$. In Table 1 we summarize the twelve studied cases with the resulting mean value for the length, in base pairs, of the linker DNA between nucleosomes that slightly depends on ϕ and α. The overall average length of the linker DNA sections is 39.6 base pairs and with values of 36.3 and 44.4 for the two extreme cases. We must remark that the predicted DNA length between histone octamers agrees with the widely reported values (17, 18, 47–49) without the need of imposing any parameter. Finally, and in order to correlate our work with experimental examples, the longest simulated chromatin corresponds to 88×10^6^ base pairs, which is approximately the size of human chromosome 16.

### Chromatin Packing Properties

**Figure 3:**
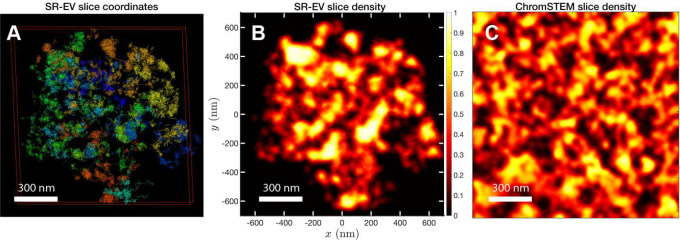
Slab images: A) representation of a 100 nm slab cut at the center of a SR-VE conformation obtained with ϕ=0.16 and α=1.10. B) 2D chromatin density corresponding to coordinates of panel A). C) ChromSTEM 2D chromatin density obtained from a 100 nm slab of a A549 cell. The 2D density color scale is the same for B) and C), and the density is normalized to its highest value in each image.

In order to start assessing whether the SR-EV model produces realistic configurations of chromatin it is necessary to bring the model to a representation similar to the results of imaging experiments. For example, ChromSTEM captures the chromatin density from a slab of 100 nm thickness. Then, we cut a similar slab from a SR-EV configuration and transform the point coordinates of the model nucleosomes to a two dimensional density that considers the nucleosomes volume. On Figure 3-A we show a representation of a SR-EV configuration as it result from the model and on Figure 3-B the collapsed two dimensional density as a colormap highlighting the porosity of the model and the emergence of chromatin packing domains. On Figure 3-C we show a ChromSTEM image for A459 cell. Since our SR-EV structures represent a single chromosome, it does not cover the full field of view of 1300 nm × 1300 nm that can be appreciated in the experimental image. However, the qualitative resemblance of the theoretical and experimental chromatin densities is stunning. The quantitative characterization of the model and its agreement with experimental results is analyzed below.

**Figure 4:**
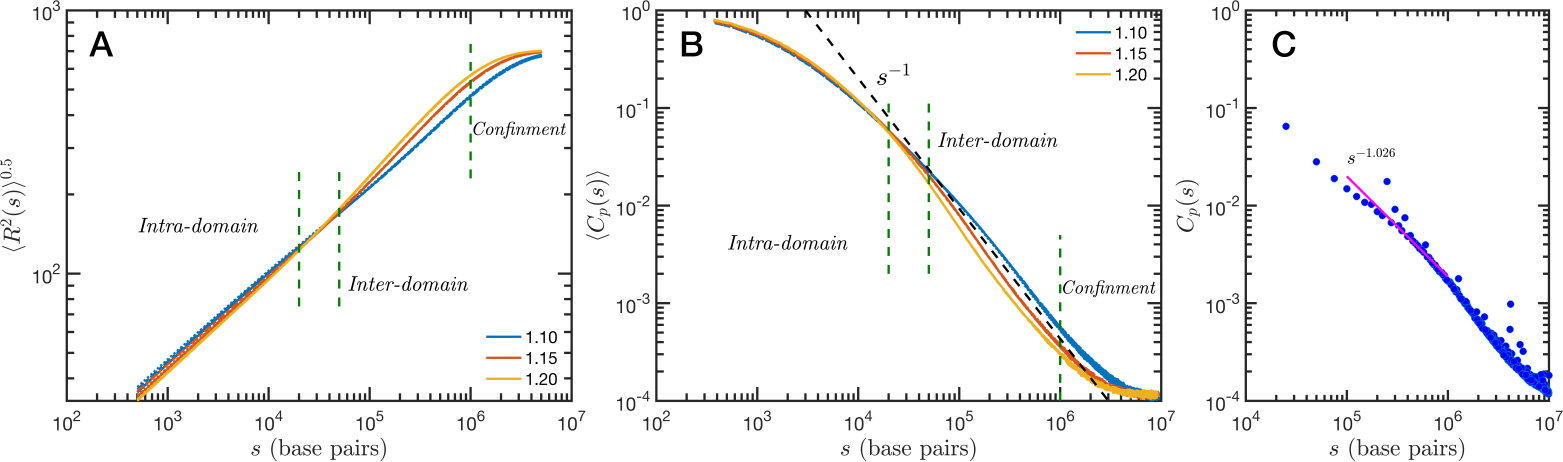
Theoretical and experimental polymeric properties of chromatin: SR-EV ensemble average of (A) end-to-end distance and (B) contact probability a as a function of the genomic distance for all simulated conditions. The crossover between short distance intra-domain and long distance inter-domain regimes is explicitly indicated, as well as the confinement effect at longer distances. Notice that on these two panels there are four lines per α value, while α∈{1.10,1.15,1.20}. (C) Experimental (Hi-C) contact probability for chromosome 1 of HCT-116 cells showing quantitative agreement with the theoretical results.

SR-EV is a non-homogeneous polymer model. The only physical interactions present in the model are the *connectivity*, the *excluded volume* and the *confinement* that, together with the *return rules* induce the formation of granular structures, or packing domains, with local density variations. This granularity can be qualitatively visualized by wrapping a mesh around the chromatin conformation, as shown in Figures 2-G and 2-H. Rotating versions of Figures 2-G and 2-H are included in the Supplementary Information as Movie M1 and Movie M2. It is worth noting that this representation is qualitatively similar to Figure 4, panels E, F and G from Ref. (50). At first glance, the wrapping interface between the region denser in chromatin and the region almost empty of chromatin resembles the dividing interface between two disordered bi-continuous liquid phases (51). We find this outcome from the SR-EV model quite interesting in view of recent claims that liquid-liquid phase separation could be related to heterochromatin and euchromatin segregation, and that chromatin domains have a liquid character (52, 53). Moreover, the bi-continuous topology offers two important functional advantages: First, the interface offers a very large surface area exposing the a significant fraction of the genome and second, the continuity of the dilute phase allows for the migration of free crowders (including proteins, transcription agents, mRNA, etc) to any region in the nucleus.

### Chromatin Polymeric Properties

The granularity of chromatin manifest itself in the polymeric properties of the model. Chromatin is a special type of polymer, and requires a careful analysis. The scaling relationship between the end-to-end distance and the polymer contour length, in this case the genomic distance, cannot be described in general with a single power law relationship, i.e. a single Flory exponent, as it is the case for synthetic polymers. In Figure 4-A we display the ensemble averaged end-to-end distance, ${\left\langle R^{2}(s)\right\rangle }^{0.5}$ as a function of the genomic distance s. All the studied cases are included in the plot, but they coalesce in three distinct groups according to the folding parameter α and with almost no effect of the overall volume fraction ϕ. The figure also shows a transition occurring for $s\sim 4\times 10^{4}$ base pairs, from a local or intra-domain regime that corresponds with distances up to 100 nm, to a long range or inter-domain one. The Flory exponent in the intra-domain regime (0.342, 0.347 and 0.354 for α=1.10,1.15 and 1.20, respectively) is consistent with a nearly space filling cluster and slightly smaller than in the inter-domain regime (0.353, 0.394 and 0.396). For s values larger than 10^6^ the curves level off due to the effect of the spherical confinement. The analysis can also be applied to the ensemble average contact probability, $\left\langle C_{p}(s)\right\rangle$, which is defined as the probability for two base pairs, separated along the polymer by a genomic distance s, of being in contact with each other (or being at a distance smaller than a cutoff). In Figure 4-B we display $\left\langle C_{p}(s)\right\rangle$ for all studied cases, using a cutoff distance of 35 nm. We see in this figure that curves depend on α but only marginally on ϕ: the four different cases for each α are almost indistinguishable in the plot. As in the end-to-end distance, here we can also distinguish a transition between intra- and inter-domain regimes. In general, the slope of $\left\langle C_{p}(s)\right\rangle$ in log-log representation is larger than −1 in the inter-domain regime, and fluctuate around −1 for inter-domain genomic distances. Figure 4-C shows the contact probability determined from Hi-C experiments. The blue dots correspond to chromosome 1 of HCT-116 cells and the behavior between 10^5^ to 10^6^ base pairs is well described by a slope very close to −1. The experimental data also show a change at intermediate separations. It is important to note to the agreement is relatively good even quantitative terms, with the transition occurring at similar genomic distance and value of $C_{p}(s)$. Since the model does not have a genomic identity or any specific architectural modifiers (e.g. CTCF and/or cohesin), the contact probability curves do not represent a particular cell or chromosome. We must mention that the other chromosomes from the HCT-116 cells have a qualitatively similar contact probability, with a power law fitting having slopes varying from −0.85 to −1.10, depending the case. The incorporation of genomic character to the SR-EV model will allow us to study all individual chromosomes contact probabilities and, more interestingly, the topological associated domains from ensembles of configurations as in Hi-C experiments.

### Chromatin Volume Concentration

**Figure 5:**
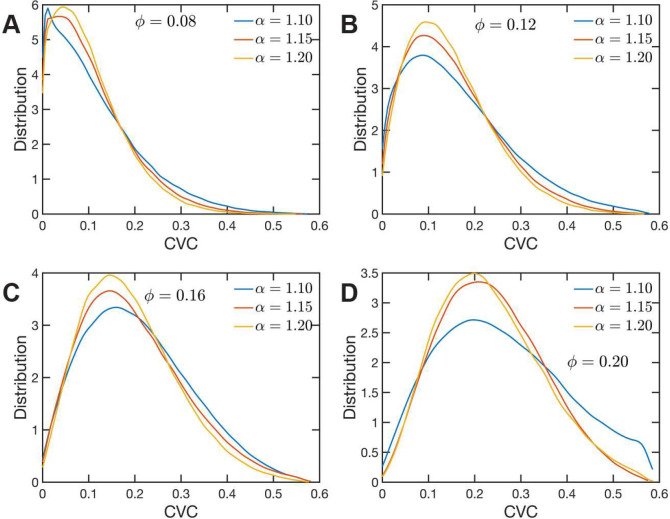
Chromatin Volume Concentration for A) ϕ=0.08, B) ϕ=0.12, C) ϕ=0.16 and A) ϕ=0.20 and α∈{1.10,1.15,1.20}. The results for ϕ=0.20,α=1.15 are the closest to the experimental findings of Ref ⁡(50). ϕ=0.08 produce CVC distributions with a much larger contribution of low density regions, and ϕ=0.20,α=1.10 over enhance the high density regions.

The heterogeneous character of chromatin revealed by experiments is captured, as we have qualitatively shown above, by the SR-EV model. A straightforward characterization of this heterogeneity is the distribution of local volume fraction calculated with a probing volume of adequate size. In the language common in chromatin experiments, this volume fraction is referred to as the Chromatin Volume Concentration (CVC) and the probing volume is, for example, a cube with an edge of 120 nm. Using electron microscopy and tomography techniques (ChromEMT) the group of Dr. Clodagh O’Shea (50) reconstructed the conformation of chromatin on a 120 nm thick slab with an area of 963 nm × 963 nm, which allowed them to measure the CVC distribution using a 8×8×1 grid with cubic cells of 120 nm edge size. To calculate the CVC from the SR-EV configuration ensembles we followed the same methodology employed in the experiments. Since we have the full 3D structure of the model chromatin we are not restricted to a slab, then we used a 6×6×6 cubic grid of (120 nm)^3^ probing volumes. Moreover, our results represent ensemble averages over the populations of 1000 replicates for each of the ϕ and α combinations. The results for each case are summarized in Figure 5 revealing that both SR-EV parameters, ϕ and α, are important in determining the CVC distributions. We see that overall the volume fraction take values up to 0.6, which is consistent with our model representing the nucleosomes as spheres that can achieve a maximum volume fraction of 0.74 as a crystal and 0.64 in the jamming limit (54). The peak of the CVC distribution increases as the overall volume fraction ϕ increases. The recent ChromEMT results reveal a CVC distribution covering a nearly identical range to our SR-EV results. Comparing with the experimental results, for the lowest overall volume fraction ϕ=0.08 the distribution has an excessive proportion of low density regions. For α=1.10 and ϕ=0.20 our results show an excess of dense regions. All other cases are qualitatively similar to the experimental results with α=1.15 and ϕ=0.20 being the closest to the experimental case.

### Packing Domains

Since the CVC is a measure using a relative large probing volume its distribution with values ranging from zero to 0.6 may be achieved by a (dynamic) smooth continuous modulation of the chromatin density or by a (also dynamic) mixing of distinct high and low density regions. The latter scheme gives rise to the concept of packing domains, as it has been recently proposed from the analysis of imaging experiments (55–57). The formation of domains is also consistent with the possibility of a microphase separation process dynamically occurring in chromatin (58–61). Moreover, a dynamic disordered bicontinuous phase separation is also in line with all the mentioned scenarios, especially considering that all imaging experiments are restricted to a quasi 2D slab of the system that could be insufficient to reveal a full 3D topology.

**Figure 6:**
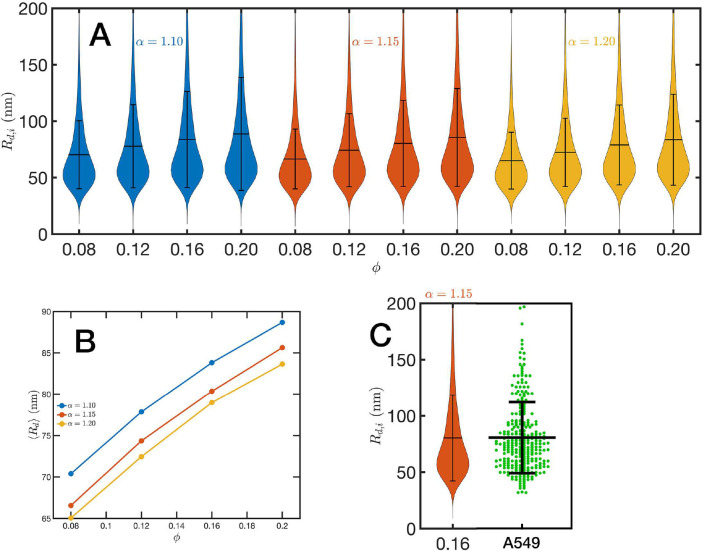
Chromatin packing domains: (A) Distributions of domain radii $R_{d,i}$ for all combinations of SR-EV parameters α and ϕ, as labeled in the figure. (B) Mean value $\left\langle R_{d}\right\rangle$ of the domain radii distributions. (C) In green, experimental distribution of domain radii obtained with ChromSTEM on A549 cell line, and the closest approximation from SR-EV that corresponds to α=1.15 and ϕ=0.16.

For the analysis of the SR-EV configurations we take advantage of the methodology developed by our experimental collaborators and transform our coordinates to a stack of images (55, 56). For this transformation each bead is represented by a normal distribution and its contribution to a given voxel of the tomogram is the integral of the normal distribution over the voxel volume. In the Supplementary Information we include Movie M3 that is an example of the resulting volumetric image stack. As we display in Figure 3-B, the image representation of the SR-EV conformations immediately reveals, in 2D, the inhomogeneity of the chromatin density that includes multiple regions of high density that we identify as packing domains. We analyzed the distribution of packing domain radii using the procedure outlined in the Supplementary Figures S1 and S2, which is essentially the same as the experimental one. In Figure 6-A we display the distribution of domain radii for all simulated conditions and the mean value for the twelve cases is displayed on Figure 6-B. For comparison, we include in Figure 6-C the results from our experiments on an A549 cell line (55) obtained with ChromSTEM that agree very well with the theoretical values in general, and in particular the agreement is excellent with the case corresponding to α=1.15 and ϕ=0.16.

### Packing Scaling Exponent D

In order to further characterize the structure of the model chromatin we calculated the pair correlation function between the model nucleosomes, i.e. g(r). From the model definition and previous analysis we know that g(r) must reveal different features at different length scales. At short distances, r≲40nm,g(r) shows the structure of the dense packing domains through the typical maxima and minima, at the intermediate distances corresponding to the average size of the packing domains and the transition between intra- and inter-domains g(r) is a decreasing function of r approaching the expected plateau for large distances. Motivated by the mass scaling analysis introduced in ChromSTEM experiments (55, 56) we will use the integral form of the pair correlation function: $G(r)=\int_{0}^{r}\;4\pi r^{\prime 2}g\left(r^{\prime }\right)dr^{\prime }$. G(r) smoothes out the short distance oscillations of g(r) and reflects the intermediate regime as a power law with exponent D<3.

**Figure 7:**
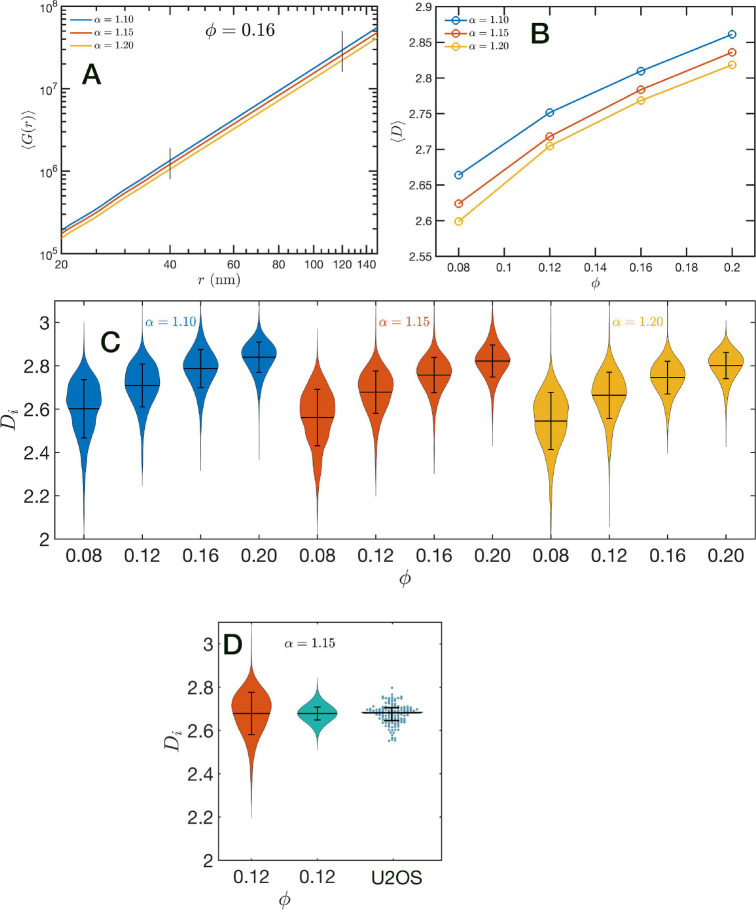
Packing coefficient D: (A) Ensemble average cumulative pair correlation function ⟨G(r)⟩ for ϕ=0.16 and the three studied values of α. The vertical black lines mark the boundaries used to perform a power law regression to calculate D. (B) Packing coefficient ⟨D⟩ as a function of ϕ and α. (C) Distribution of packing coefficient $D_{i}$ for all the individual configurations for the twelve simulated conditions. (D) Effect of resampling of distribution of $D_{i}$ by replacing groups of 10 measurements by a their mean value and comparison with experimental PWS D values for U2OS cells. The mean value agrees very well with the SR-EV results for ϕ=0.12 and α=1.15. The width of the distribution reflects that the experimental value were calculated as an average number representing the whole (or a large region of the) nucleus.

In Figure 7-A we show in a log-log representation, as an example, the ensemble average ⟨G(r)⟩ corresponding to the global volume fraction ϕ=0.16 and the three values of α. Between 40 nm and 120 nm we found that the ⟨G(r)⟩ is essentially a perfect straight line, i.e. $\langle G(r)\rangle \propto r^{D}$. We define D as the packing parameter that we calculate for 40<r/nm<120. The slopes for the three displayed cases are slightly different, with D values ranging between 2.75 to 2.80 as α decreases from 1.20 to 1.10. In Figure 7-B we summarize the results for D for all the simulated conditions, which shows that D has a positive correlation with ϕ, the overall volume fraction of the whole configuration, and a weaker inverse dependence on the folding parameter α.

A similar power law regression can be applied on the $G_{i}(r)$ obtained for each configuration. We use the subscript i to distinguish that the quantity corresponds to a single configuration i. Since the configurations are obtained using a stochastic procedure, there is a large variability in the power law fits obtained from them and some examples are included in Figure S3 of the Supplementary Information. In Figure 7-C we show the distributions of $D_{i}$ values for all twelve simulated conditions. Notice that individual $D_{i}$ can be larger than 3. For comparison, we include in Fig 7-E an experimental result obtained with PWS experiments on U2OS cells. The spread of the distribution is relatively large and reflect the size of the system over which $G_{i}(r)$ is calculated. In the SR-EV case, this is a sphere of 240 nm radius. This is important for the comparison with experiments obtained from PWS that collect information on a full cross section of the nucleus. Consequently, the spread of the PWS distribution of D values is narrower than the one that we get from our current SR-EV configurations. In order to compensate for the different size of the sampling region we reduced the ensemble by grouping 10 consecutive $D_{i}$ values into a single $D_{i}^{*}$ that is the mean value over those 10 values. The resulting distribution is displayed on Figure 7-D that compares very well with the experimental one in both, the mean value and the spread of the distribution.

**Figure 8:**
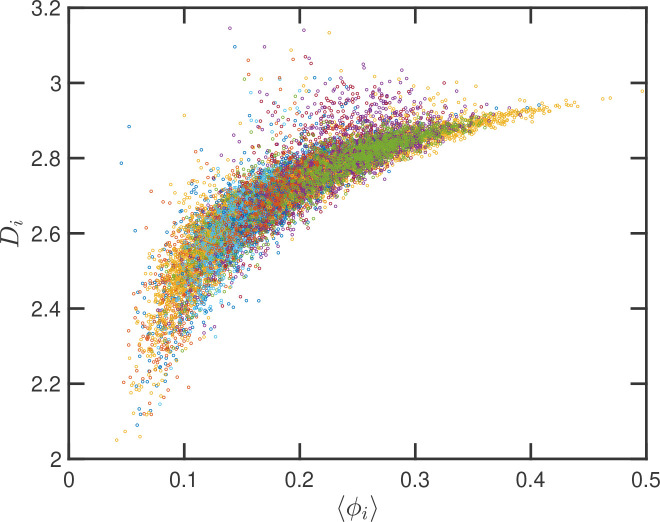
Local correlation between packing parameter and chromatin volume concentration: Relation between the calculated $D_{i}$ with the average local volume fraction $\left\langle \phi_{i}\right\rangle$. Both quantities are calculated for the same configuration and in the same spherical region of 240 nm in radius. The figure includes one point for each one of the 12,000 configurations of the twelve simulated ensembles.

Up to this point we have performed our analysis based on the SR-EV parameters α and ϕ to distinguish the different ensembles of configurations. However, the local volume fraction, as it has been shown above in Figure 2 and Figure 5, fluctuates at the scale of the packing domain size. This inhomogeneity makes the representation of a configuration by its overall SR-EV parameter ϕ not completely meaningful when we study a local or mesoscopic property, such as the packing parameter $D_{i}$. Therefore it is convenient to introduce the local average chromatin volume fraction $\left\langle \phi_{i}\right\rangle$ calculated in exactly the same 240 nm sphere that we use to calculate $D_{i}$. The correlation between these two mesoscopic quantities is plotted in Figure 8 and includes every one of the SR-EV 12,000 configurations. There is a very clear and interesting correlation between $D_{i}$ and $\left\langle \phi_{i}\right\rangle$. For high $\left\langle \phi_{i}\right\rangle$ the local $D_{i}$ approaches to 3, which is the theoretical upper limit for ⟨D⟩. For intermediate and small $\left\langle \phi_{i}\right\rangle$ there is a quite wide distribution of $D_{i}$ values, consistent with the violin plots of Figure 7-C. Nevertheless, the local chromatin volume fraction is the main factor determining the corresponding packing parameter.

## Discussion

We present a novel model of chromatin based on stochastic returns and physical interactions that captures the ground truth structures observed across both imaging and sequencing based measures of chromatin organization. By maintaining in SR-EV the possibility of self-returning extensions that are presented in SRRW, several features arise. (1) High frequency, short return events lead to the formation of individual packing domains. (2) Low frequency, large steps give rise to a corrugated chromatin structure at intermediate length scales (~ 100 nm) that allows genomic accessibility to arise (Figure 2). Expanding on the theory originally presented by Huang et al. (45), we now can account for excluded volume interactions between single nucleosomes to quantitively and qualitatively represent chromatin configurations. This extension is crucial as it allows for the accurate reconstruction of the occupied volumes within chromatin and to calculate the physical properties of genomic organization. Pairing the excluded volume representation of the individual monomer units (nucleosomes) with stochastic returns produces a continuous heterogeneous polymer chain with a random distribution of space-filling domains. In comparing the effects of the folding parameter, α, with the overall chromatin volume fraction, ϕ, we show that just two parameters can recapture the heterogeneous nature of chromatin observed in electron microscopy, the variations in chromatin volume concentrations, the formation of packing domains with appropriate sizes, that power-law distributions are present at intermediate length scales (quantified by D), and the heterogeneity observed experimentally in live cell measurements of chromatin structure.

Crucially, the SR-EV model is grounded in the stochastic description of genome organization which allows capturing both the description of ensemble properties (e.g., populations of cells/chromosomes) and individual chromosomes. This feature is what allows both the accurate representation of individual experiments (such as the visualized 3D structure in ChromSTEM) as well as features that only become apparent over numerous realizations (such as contact scaling observed in Hi-C, population heterogeneity observed in PWS Microscopy). The model unit length coincides with the size of a nucleosome and owing to physical principles, the linker unit produced is concordant with reported experimental values of 35–45 bp, (Table 1). The present length of the model polymer is comparable with the size of human chromosome 16 or smaller; but could be expanded with additional computational resources. Therefore, the SR-EV configurations span over a large range of spatial dimensions (~10 nm – ~1 μm). The agreement with the experimentally found CVC distributions gives us a first confirmation on the validity of the model, and an indication of the relevant values for α and ϕ present physiologically. The quantitative agreement of the packing domain radii distribution with the outcome of ChromSTEM reinforce the confidence in the theory. The packing parameter D is defined in terms of the incremental pair correlation function between model nucleosomes; a definition that is similar (but not exactly the same) as the one proposed in ChromSTEM studies. The value of D is consistently found between 2 and 3 for all simulated conditions. D is calculated on a mesoscopic region of 240 nm in radius, which is completely independent of the location of the packing domains. However, since we show that there is a strong positive correlation between $D_{i}$ and the corresponding local volume fraction $\left\langle \phi_{i}\right\rangle$ we can infer that regions containing large packing domains will be associated with a large D. The distribution of $D_{i}$ values span over the same range of values observed in PWS experiments. In particular, we show a case in excellent quantitative agreement with PWS results for U2OS cell line (noting that similar distributions are observed independently of this cancer cell line).

Finally, we view the simplicity of our model as a core strength as it already captures key details about genome organization without introducing many of the constraints present within existing frameworks. Currently, we could generate 12,000 independent configurations of a 500,000 nucleosome (75 Mbp, approximately the size of chromosome 16) within a short period of time. Likewise, we envision that future work can incorporate some of the myriad molecular features known to exist within chromatin organization to be able to interrogate how key components (e.g. sparse, focal constraint such as CTCF binding sites or heterochromatin modifying enzymes) would alter the observed physical structures. As with any modeling work, there will always be the tension between the addition of details for fidelity and the ability to capture the properties of genome organization. As the SR-EV already captures many key properties seen within chromatin, we anticipate that it can serve as the basis model of stochastically configured genome organization within the wider field.

## Materials and Methods

### Cell Culture

Human cell line U2OS cells (ATCC, #HTB-96) used for experimental validation of the model were cultured in McCoy’s 5A Modified Medium (Thermo Fisher Scientific, #16600–082) supplemented with 10% Fetal bovine serum (FBS) (Thermo Fisher Scientific, #16000–044) and 100 μg/ml penicillin-streptomycin antibiotics (Thermo Fisher Scientific, #15140–122). Human cell line A549 cells (ATCC, #CCL-185) used for experimental validation of the model were cultured in Dulbecco’s Modified Eagle’s Medium (Thermo Fisher Scientific, #11965092) supplemented with 10% Fetal bovine serum (FBS) (Thermo Fisher Scientific, #16000–044) and 100 μg/ml penicillin-streptomycin antibiotics (Thermo Fisher Scientific, #15140–122). Experiments were performed on cells from passages 5–10. All cells were maintained under recommended conditions at 37°C and 5% CO_2_. Cells were verified to have no detectable mycoplasma contamination (ATCC, #30–1012K) prior to starting experiments.

### PWS Sample Preparation

Prior to imaging, cells were cultured in 35 mm glass-bottom petri dishes. All cells were allowed a minimum of 24 hours to re-adhere and recover from trypsin-induced detachment. PWS imaging was performed when the surface confluence of the dish was approximately 70%.

### PWS Imaging

The PWS optical instrument consists of a commercial inverted microscope (Leica, DMIRB) equipped with a broad-spectrum white light LED source (Xcite-120 light-emitting diode lamp, Excelitas), 63x oil immersion objective (Leica HCX PL APO, NA1.4 or 0.6), long pass filter (Semrock, BLP01–405R-25), and Hamamatsu Image-EM CCD camera C9100–13 coupled to an LCTF (CRi VariSpec). Live cells were imaged and maintained under physiological conditions (37°C and 5% CO_2_) using a stage top incubator (In Vivo Scientific, Stage Top Systems). Briefly, PWS directly measures the variations in spectral light interference that results from internal light scattering within the cell, due to heterogeneities in chromatin density, with sensitivity to length scales between 20 and 300 nm (56). Variations in the refractive index distribution are characterized by the mass scaling (chromatin packing scaling) parameter, D. A detailed description of these methods is reported in several publications (43, 44, 62, 63)

### ChromSTEM Sample Preparation and Imaging

Cell samples were prepared as reported in (55). Cells were first washed with Hank’s Balanced Salt Solution (HBSS) without calcium and magnesium (Thermo Fisher Scientific, #14170112) 3 times, 2 minutes each. Fixation, blocking, DNA staining and DAB solutions were prepared with 0.1 M sodium cacodylate buffer (pH=7.4). Cells were fixed with 2% paraformaldehyde, 2.5% glutaraldehyde, 2 mM calcium chloride for 5 minutes in room temperature and 1 hour on ice and all the following steps were performed on ice or in cold temperature unless otherwise specified. After fixation, cells were washed with 0.1 M sodium cacodylate buffer 5 times, 2 minutes each. Cells were then blocked with 10 mM glycine, 10 mM potassium cyanide for 15 minutes. Cells were stained with 10 μM DRAQ5, 0.1% Saponin for 10 minutes and washed with the blocking solution 3 times 5 minutes each. Cells were bathed in 2.5 mM 3,3’-diaminobenzidine tetrahydrochloride (DAB) and exposed to 150 W Xenon Lamp with 100x objective lens and a Cy5 filter for 7 minutes. Cells were washed with 0.1 M sodium cacodylate buffer 5 times, 2 minutes each, followed by staining with 2% osmium tetroxide, 1.5% potassium ferrocyanide, 2 mM calcium chloride, 0.15 M sodium cacodylate buffer for 30 minutes. After osmium staining, cells were washed with double distilled water 5 times, 2 minutes each and sequentially dehydrated with 30%, 50%, 70%, 85%, 95%, 100% twice, ethanol, 2 minutes each. Cells were then washed with 100% ethanol for 2 minutes and infiltrated with Durcupan^™^ ACM ethanol solutions (1:1 for 20 minutes, and 2:1 for 2 hours) at room temperature. Cells were then infiltrated with resin mixture for 1 hour, resin mixture with accelerator for 1 hour in 50°C dry oven and embedded in BEEM capsule with the resin mixture at 60°C dry oven for 48 hours.

Resin sections with thickness around 100 nm were prepared with a Leica UC7 ultramicrotome and a 35°C DiATOME diamond knife. The sections were collected on copper slot grids with carbon/Formvar film and 10 nm colloidal gold nanoparticles were deposited on both sides of the section as fiducial markers. HAADF images collected by a 200 kV cFEG Hitachi HD2300 scanning transmission electron microscope. For each sample, projections were collected from −60°C to +60°C with 2°C increments, along 2 roughly perpendicular axes.

Each projection series along one rotation axis was aligned with IMOD using gold nanoparticle fiducial markers. After image alignment, penalized maximum likelihood algorithm in Tomopy was used to reconstruct the images with 40 iterations. IMOD was used to combine tomograms from different rotation axis of the same sample.

### Chromatin Domain Radius Measured from Experiment

The chromatin domains were identified using FIJI. 2D chromatin density distributions were obtained by re-projection of the tomogram along *z*-axis, followed by Gaussian filtering with 5 pixels radius and CLAHE contrast enhancements with block size of 120 pixels. Chromatin domain centers were selected as the local maxima of chromatin density.

To evaluate the size of a domain, 2 properties were analyzed for each domain, which are the mass scaling properties and radial volume chromatin concentration (CVC). For mass scaling, multiple mass scaling curves were sampled by using pixels (a 11-pixel × 11-pixel window) around the center of an identified domain and they were averaged by the weight of the pixel values of the selected center pixel. A size of domain is defined by the length scale that the domain meets any of the following 3 criteria: (i) It deviates from the power-law mass scaling relationship $M(r)\propto r^{D}$ by 5%; (ii) The local fitting of D reaches 3; (iii) The radial CVC reaches a local minimum and begins to increase for longer length scale.

### Experimental Validation Plots

GraphPad Prism 10.0.0 was used to make the violin plots in Figure 5F and Figure 6E. The violin plots are represented as individual data points, with lines at the median and quartiles.

## Figures and Tables

**Table 1:** Linker DNA mean value for the twelve ϕ, α studied combinations. The folding parameter α controls the return rules, Eqs. (1) and (2). *N* is the total number of nucleosomes represented in the model, which is related to the overall volume fraction $\phi =N{\left(r_{\circ }/R_{c}\right)}^{3}$ with $r_{\circ }$ representing the radius of the nucleosomes and $R_{c}$ the global spherical cutoff. The average number of DNA base pairs per model nucleosome, including the linker DNA, is 186.6.

*Mean value of linker DNA length (bp)*				
*ϕ*	*_N_╲^α^*	***1.10***	***1.15***	***1.20***
0.08	186741	40.8	38.0	36.3
0.12	280112	41.8	38.6	36.6
0.16	373483	44.4	39.4	37.4
0.20	466854	43.2	42.0	36.9
